# Syphilis Self-Testing Among Female Sex Workers in China: Implications for Expanding Syphilis Screening

**DOI:** 10.3389/fpubh.2022.744240

**Published:** 2022-04-13

**Authors:** Cheng Wang, Xia Li, Yajie Wang, Bin Yang

**Affiliations:** ^1^Dermatology Hospital of Southern Medical University, Guangzhou, China; ^2^Southern Medical University Institute for Global Health and Sexually Transmitted Diseases, Guangzhou, China

**Keywords:** syphilis, testing, female sex worker (FSW), experience, determinant

## Abstract

**Background:**

Syphilis self-testing (SST) may help expand syphilis test uptake among female sex workers. However, there has been no studies on examining SST among female sex workers. We aim to examine SST experience and its determinants among female sex workers in China.

**Methods:**

A venue-based, cross-sectional study of female sex workers was conducted in eight Chinese cities in 2019. Participants completed a survey including socio-demographic characteristics, sexual behaviors, and SST history. Multivariable logistic regression was conducted to evaluate the associated factors with SST.

**Results:**

Among 1,287 Chinese female sex workers, 72.1% (928/1,287) had ever tested for syphilis, and 5.9% (76/1,287) had ever used syphilis self-testing. Among syphilis self-testers, more than half (57.9%, 44/76) reported that the self-test was their first syphilis test, around one-fifth (18.4%, 14/76) reported that syphilis self-testing results influenced the price of commercial sex. After adjusting for covariates, female sex workers who received anal sex in the past month (adjusted odds ratio [aOR]: 2.6, 95%CI: 1.5–4.3, *p* < 0.001), used drugs before or during sex (aOR: 3.8, 95%CI: 2.3–6.4, *p* < 0.001), tested for other sexually transmitted infections (STIs) in the past 6 months (aOR: 3.4, 95%CI: 1.9–6.0), ever tested in the hospital (aOR: 5.1, 95%CI: 2.5–10.4, *p* < 0.001), and ever tested in the community (aOR: 1.7, 95%CI: 1.3–2.2, *p* < 0.001) were more likely to perform syphilis self-testing.

**Conclusions:**

Syphilis self-testing has the potential to expand testing coverage, and increase testing frequency with limited potential harms among FSW. Further evaluation on the intervention effects based on syphilis self-testing among FSW are needed.

## Background

Syphilis continues to be a major public health concern globally, with an estimation of around 6 million new cases of syphilis occurring every year among adults aged between 15 and 49 years in the world (1). Female sex workers are far more likely to be infected with syphilis than people in the general population (2), around 15 countries reported more than 5% prevalence of syphilis in the female sex workers worldwide (3). Regular syphilis testing is a key strategy for syphilis control among female sex workers, as well as their clients, spouses and fetus (4, 5). However, syphilis testing uptake remains low among female sex workers in low- and middle- income countries (LMIC) (6). Studies suggest that approximately half of Chinese female sex workers remain unaware of syphilis serostatus (7).

Currently, syphilis testing among female sex workers mainly depends on facility-based services such as voluntary counseling and testing (VCT) clinics, but existing facility-based syphilis testing and management resources are limited and it highly dependent on active participation (8). Obviously, it is not friendly to vulnerable FSWs who are unfamiliar with testing methods and fear stigma associated with syphilis (9–12). Moreover, inconvenient testing systems, lack of privacy, and the recent COVID-19 related restrictions make facility-based testing more difficult (9–12).

Syphilis self-testing may help improve test uptake among female sex workers. Syphilis self-testing is the process whereby a person collects his/her own blood, performs the test, and interprets the result themselves (9). An immunochromatographic test uses blood to detect treponemal antibodies using a rapid test, similar to blood-based HIV self-testing (9). Preliminary research conducted by the Netherlands (13) and our team (9) has shown that the syphilis self-testing is highly accepted in MSM, with obvious benefits and limited harms. New evidence from our recent randomized controlled trial (RCT) showed that self-testing for syphilis can significantly increase the MSM detection rate (9, 14). Yet, there is no literature on syphilis self-testing among female sex workers.

The purpose of this study was to examine syphilis self-testing experience and its determinants among female sex workers in China.

## Methods

### Study Design and Participants

**Figure 1:**
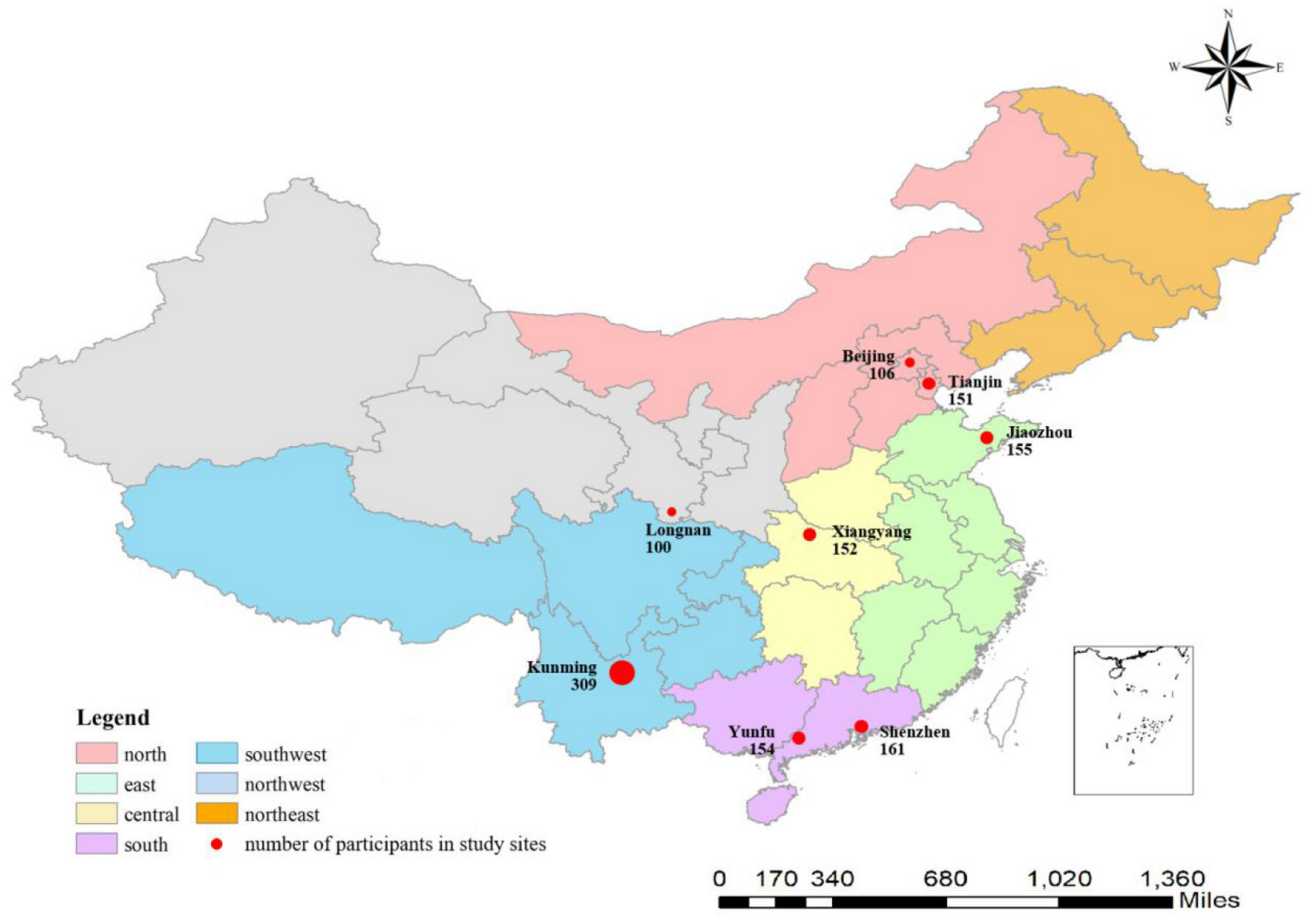
Participants geographic distribution.

We conducted a venue-based, cross-sectional study in eight cities (Beijing, Tianjin, Shenzhen, Kunming, Jiaozhou, Yunfu, Xiangyang, and Longnan) in Chinese seven provinces between August 17 and October 17, 2019 (Figure 1). These eight cities were selected based on local capacity and the availability of ongoing public health outreach programs for female sex workers, which covered six major regions of China (North, Northwest, Southwest, Central South, East and South).

We partnered with eight local community-based organizations (CBO) that have years of experience in sexually transmitted diseases (STD) intervention and outreach for female sex workers, including condom promotion, sexual health education, HIV and syphilis rapid testing and counseling, and linkage to care (subsequent clinical services for infected individuals). Prior to this study, the sampling frame was generated based on a map of commercial sex venues created by local CBO in each study site according to geographic area and type of venues. A convenience sampling method was used to recruit eligible participants.

Female sex workers were categorized into two tiers (high- and low-tier) based on the clientele's socioeconomic status (15). Low-tier venues include foot bathing shops, hair salons or barber shops, massage parlors, roadside restaurants, roadside shops, guesthouses, streets or outdoor public places. High-tier venues include karaoke bars, hotels, sauna, and nightclub. At least 30% of total female sex workers were recruited from low-tier venues at each site.

All eligible participants were required to meet the following criteria: born biologically female; aged 18 or above; earn money or goods through commercial sex (vaginal, oral, and/or anal) at least once in the past 3 months; volunteered to participate in the survey and signed an informed consent.

### Data Collection

An online survey link was created by using the Wenjuanxing, an e-questionnaire platform (Changsha Haoxing Information Technology Co., Ltd., Changsha, China), which allowed participants to complete the survey with smartphone or computer tablets. The survey questionnaire and procedures were designed based on discussions with the local CBO stakeholders, policy makers and international HIV/STI experts. A pilot survey among 50 female sex workers was conducted to simplify the survey administration and improve the comprehensibility. The pilot data was not included in the final analysis.

In the formal survey, interested participants who clicked on the survey link were required to sign an online informed consent by clicking on a “button” indicating that the participant has read the consent form and agrees to participate. Eligible participants then self-administered the survey, with the assistance of outreach workers if needed. To avoid repeated participation, each participant's mobile phone number was verified with a code. The surveys completed within 5 min were deemed invalid according to our pilot study results. Participants were provided with $2 USD for their time for completing the survey.

### Syphilis Self-Testing Kits in China

Syphilis self-testing kits can be accessed on e-commence platforms or through existing HIV self-testing programs in China. More than 10 brands of syphilis self-testing kits are available on the two largest e-commerce platforms in China (Taobao and Jingdong). All the kits used an immune colloidal gold technology to detect Treponema pallidum specific antibodies in the blood, which cannot distinguish between current and past infection. All the kits were approved by the China Food and Drug Administration with an excellent sensitivity and specificity. The cost per test kit ranges from 2·5 to 15 US dollars (9).

### Measures

We asked about the syphilis self-testing history through the following questions: location where the kit was obtained, whether the individual performed self-testing alone or with a sexual partner, self-test results, post-test healthcare seeking behaviors, subsequent changes in syphilis testing frequency after initial use of a self-test kit, potential harms of self-testing, influences of performing self-testing on sex exchange, and experiences of being pressured to take a syphilis self-test.

The survey also asked about: 1) social-demographic characteristics, including: age, marital status, place of residence, annual income, and education level. 2) Sexual behaviors: time of providing commercial sex in the current location, number of cities worked for selling sex, number of clients served in the past month, charge for vaginal sex, whether condoms were used consistently when engaged in commercial sex in the past month, illicit substance use, STI testing history. Consistent condom use was defined as always using a condom during commercial sex.

### Statistical Analysis

We conducted a descriptive analysis on sociodemographic information, sexual behavior, and syphilis self-testing history. Univariable and Multivariable logistic regression was conducted to explore associated factors with syphilis self-testing. The variables of age, legal marital status, educational attainment, and annual income were adjusted in the multivariable model. In addition, a sub-analysis was performed to explore the correlates of syphilis self-testing among participants who had ever used HIV self-testing. Statistical significance was defined as *P* < 0.05. All analyses were performed using SAS (V9.2, SAS Institute Inc., Cary, NC).

### Ethics

This study was approved by the Ethics Committee of the Dermatology Hospital of Southern Medical University (2019017).

## Results

A total of 1,287 female sex workers completed the survey and were included in the final analyses. Among them, 928 women (72.1%, 928/1,287) had ever tested for syphilis while 76 participants (6.0%, 76/1,287) had ever used syphilis self-testing.

### Participant's Characteristics

Most participants were between 18 and 35 years old (53.8%, 1,043/1,287), being married (43.8%, 564/1,287), had a junior high school degree (45.2%, 582/1,287), had an annual income between USD $5,001 and $14,000 (56.3%, 725/1,287), being from low tier venues (57.3%, 738/1,287), being employed by a boss (55.6%, 715/1,287), being a resident in the sampling province (51.4%, 662/1,287), and working in the current province for more than 1 year (50.5%, 650/1,287; Table 1).

**Table 1:** Social demographic characteristics among female sex workers, a cross-sectional survey in China, 2019.

**Characteristics**	**Total**	**Syphilis testing (%)**		
		**Self-tester**	**Facility-tester**	**Non-tester**
**Total**	1,287	76 (5.9)	852 (66.2)	359 (27.9)
**Age**				
18~25	271 (22.5)	7 (9.6)	206 (25.8)	58 (17.6)
26~35	376 (31.3)	22 (30.1)	243 (30.4)	111 (33.6)
36~45	312 (25.9)	25 (34.3)	185 (23.1)	102 (30.9)
>45	244 (20.3)	19 (26.0)	166 (20.7)	59 (17.9)
**Workplace**				
High tier	549 (42.7)	33 (43.4)	391 (45.9)	125 (34.8)
Low tier	738 (57.3)	43 (56.6)	461 (54.1)	234 (65.2)
**Legal marital status**				
Never married and not cohabiting	227 (17.6)	13 (17.1)	146 (17.1)	68 (18.9)
Never married but cohabiting	167 (12.9)	6 (7.9)	129 (15.1)	32 (8.9)
Married	564 (43.8)	31 (40.8)	355 (41.7)	178 (49.6)
Divorced or widowed	329 (25.6)	26 (34.2)	222 (26.1)	81 (22.6)
**Highest education**				
Elementary school or below	481 (37.4)	30 (39.5)	308 (36.2)	143 (39.8)
Junior high school	582 (45.2)	26 (34.2)	378 (44.4)	178 (49.6)
Senior High school or above	224 (17.4)	20 (26.3)	166 (19.5)	38 (10.6)
**Annual income (USD)**				
< $2,000	12 (0.9)	0 (0.0)	11 (1.3)	1 (0.3)
$2,001~$5,000	202 (15.7)	13 (17.1)	84 (9.9)	105 (29.3)
$5,001~$9,000	398 (30.9)	29 (38.2)	239 (28.0)	130 (36.2)
$9,001~$14,000	327 (25.4)	15 (19.7)	231 (27.1)	81 (22.5)
>$14,000	348 (27.0)	19 (25.0)	287 (33.7)	42 (11.7)
**Employed status**				
Self-employed	572 (44.4)	34 (44.8)	371 (43.5)	167 (46.5)
Employed by a boss	715 (55.6)	39 (55.2)	474 (56.5)	187 (53.5)
**Length of time working in current location**				
0~6 months	383 (29.8)	22 (28.9)	184 (21.6)	177 (49.3)
7~12 months	254 (19.7)	4 (5.3)	167 (19.6)	83 (23.1)
Over 1 year	650 (50.5)	50 (65.8)	501 (58.8)	99 (27.6)
**Number of cities worked in for selling sex**				
1	730 (56.7)	48 (63.2)	493 (57.8)	189 (52.6)
2	261 (20.3)	14 (18.4)	194 (22.8)	53 (14.8)
3	110 (8.6)	2 (2.6)	85 (10.0)	23 (6.4)
>3	186 (14.5)	12 (15.8)	80 (9.4)	94 (26.2)
**Residence**				
Sampling province	662 (51.4)	46 (60.5)	446 (52.4)	170 (47.4)
Other province	625 (48.6)	30 (39.5)	406 (47.6)	189 (52.6)

*USD, United States dollar*.

### Sexual Behaviors

Among 1,287 women, the average number of clients served in the past month was 22 (IQR: 12–56). The average amount of payment received for vaginal sex was USD $20 (IQR: 15–45). A minority of female sex workers used condoms consistently when engaged in vaginal sex (42.1%, 542/1,287), oral sex (16.0%, 120/750) and anal sex (41.6%, 101/243) with clients in the past month, respectively. Most women reported having never used drugs before or during sex (78.4%, 1,009/1,287). Over half reported bulk purchasing of condoms (59.7%, 768/1,287), and quite few individuals reported bulk purchasing of syphilis self-testing kits (5.2%, 67/1,287). Almost all women reported having ever received HIV/STD related services in the past year (95.7%, 1,232/1,287). A total of 1,072 women (83.3%, 1,072/1,287) had ever tested for HIV, and 103 (8%, 103/1,287) had ever used HIV self-testing. Around half of individuals reported ever testing for other STDs (herpes, chlamydia, gonorrhea, warts and hepatitis) in the past 6 months (50.2%, 646/1,287; Table 2).

**Table 2:** Sexual behavior characteristics among female sex workers, a cross-sectional survey in China, 2019.

**Variables**	**Total**	**Syphilis testing (%)**		
		**Self-tester**	**Facility-tester**	**Non-tester**
Total*	1,287	76 (5.9)	852 (66.2)	359 (27.9)
**Number of clients served in the past month, median (IQR)**				
	22 (12–56)	15.5 (8–45)	22 (12–60)	22 (10–50)
**Received money for vaginal sex (USD), median (IQR)**				
	20	30	25	20
	(15–45)	(15–45)	(15–45)	(100–30)
**Consistently used condom in commercial vaginal sex in past month**				
	542 (42.1)	36 (47.4)	368 (43.1)	138 (38.4)
**Provided oral sex in the past month**				
	750 (58.3)	47 (61.8)	521 (61.1)	182 (50.7)
**Consistently used condom in commercial oral sex in past month**				
	120 (16.0)	7 (14.9)	79 (15.1)	34 (18.7)
**Provided anal sex in the past month**				
	243 (18.9)	25 (32.9)	159 (18.7)	59 (16.4)
**Consistently used condom in commercial anal sex in past month**				
	101 (41.6)	10 (40.0)	63 (39.6)	28 (47.5)
**Used drugs before or during sex**				
	278 (21.6)	32 (42.1)	169 (19.8)	77 (21.4)
**Injecting drugs in the past 6 months**				
	56 (4.3)	3 (3.9)	43 (5.1)	10 (2.8)
**Received any kind of HIV/STD-related services in the last year**				
	1,232 (95.7)	73 (96.1)	842 (98.8)	317 (88.3)
**Bulk purchase of condoms**				
	768 (59.7)	38 (50.0)	540 (63.4)	190 (52.9)
**Bulk purchase of HIV/syphilis self-test kits**				
	67 (5.2)	22 (28.9)	28 (3.3)	17 (4.7)
**Tested for other STIs in the past 6 months**				
	646 (50.2)	57 (75.0)	495 (58.1)	94 (26.2)
**Diagnosed with other STIs**				
	394 (30.6)	19 (25.0)	303 (35.6)	72 (20.1)
**Ever had HIV testing**				
	1,072 (83.3)	72 (94.7)	842 (98.8)	158 (44.0)
**Ever had HIV self-testing**				
	103 (8.0)	52 (68.4)	42 (4.9)	9 (2.5)
**Ever tested in the hospital**				
	692 (53.8)	65 (85.5)	542 (63.6)	-
**Ever tested in the community**				
	794 (61.7)	47 (61.8)	647 (75.9)	-

*IQR, interquartile range; STI, sexual transmitted infection*.

### Syphilis Self-Testing Experience

Among the 76 women who had self-tested for syphilis, more than half (57.9%, 44/76) reported that the self-test was their first ever syphilis testing, and most (81.6%, 62/76) reported that the self-testing was undertaken in conjunction with HIV self-testing. About one-third of women (35.5%, 27/76) reported an increase in the frequency of syphilis testing after initial use of a self-test kit. A minority of women reported even giving (18.4%, 14/76) or selling (1.3%, 1/76) self-test kits to their clients. The most common site to obtain a syphilis self-test kits was from a community-based organization (82.9%, 63/76), followed by online drug stores (28.9%, 22/76; Table 3).

**Table 3:** Past syphilis self-test experience, post-test health services utilization, and potential harms of syphilis self-testing among Chinese female sex workers.

**Attributes**	**Syphilis self-tester (*N* = 76, %)**
**Characteristics of self-testing**	**76**
**Location where self-test kit was obtained**	
Community-based organization	63 (82.9)
Online drug store	22 (28.9)
Hospital	21 (27.6)
Friend	7 (9.2)
Pharmacy	5 (6.6)
**Self-testing results (last self-test)**	
Reactive	13 (17.1)
Not sure	10 (13.1)
Negative	53 (69.7)
**Post-test actions**	**23**
Sought care following reactive/uncertain self-testing result	20 (86.9)
**Time since reactive/uncertain self-testing result to seeking care**	**20**
0–2 weeks	16 (80.0)
2–4 weeks	-
1–3 months	2 (10.0)
>3 months	2 (10.0)
**Location for seeking care**	**20**
General hospital	4 (20.0)
Specialist STI service	8 (40.0)
Center for Disease Control and Prevention	7 (35.0)
Pharmacy/ Online counseling/others	1 (5.0)
**Benefits**	**76**
Self-test as their first-time test	44 (57.9)
Gave a self-test kit to a client	14 (18.4)
Sold a self-test kit to a client	1 (1.3)
Joined with HIV self-testing	62 (81.6)
Increased testing uptake after first self-test	27 (35.5)
**Adverse events**	**76**
HIV/STI self-testing influenced sex pricing negotiation	14 (18.4)
Police kept self-test kits as the evidence to accuse you of selling sex	2 (2.6)
Pressured self-testing	9 (11.8)
**Types of pressure**	**76**
Physical violence	5 (6.6)
Threats of violence	2 (2.6)
Verbal abuse	3 (3.9)
Psychological pressure	5 (6.6)
Excessive control of activities	2 (2.6)
Withholding of household resources	3 (3.9)
Threatening to end a relationship	4 (5.3)

*STI, sexual transmitted infection*.

A minority of women reported a reactive (17.1%, 13/76) or uncertain (13.2%, 10/76) result in their most recent syphilis self-test, and most of them (86.9%, 20/23) sought care following syphilis self-testing. The majority (80%, 16/20) sought care within 2 weeks of self-testing, and three-quarters (75%, 15/20) sought care either at a specialty sexually transmitted infection (STI) clinic or in a Center for Disease Control and Prevention.

Among the 76 syphilis self-testers, a small proportion (18.4%, 14/76) reported that syphilis self-testing results influenced the price of sex, and 9 women (11.8%, 9/76) reported ever experiencing pressure to undertake syphilis self-testing. The most common types of pressure were physical violence (6.6%, 5/76), psychological stress (6.6%, 5/76), and threatening to end a relationship (5.3%, 4/76; Table 3).

### Motivations and Barriers to Syphilis Self-Testing

Most syphilis self-testers (73.68%, 56/76) reported some difficulties in performing self-testing, including collecting blood with a tube (53.6%, 30/56) or pricking fingers (50%, 28/56). The most commonly reported motivations for syphilis self-testing were that they wanted to know their infectious status (60.5%, 46/76), and they recently had high risk contact (55.2%, 42/76). The most common reason for not using self-testing was that they have never heard of syphilis self-testing before (36.8%, 446/1,211; Supplementary Table S1).

### Factors Associated With Syphilis Self-Testing

After adjusting for age, legal marital status, educational attainment and annual income, the multivariable regression model showed that the following factors were positively associated with syphilis self-testing: receiving anal sex in the past month (adjusted odds ratio [aOR]: 2.6, 95%CI: 1.5–4.3, *p* < 0.001), using drugs before or during sex (aOR: 3.8, 95%CI: 2.3–6.4, *p* < 0.001), tested for other sexually transmitted infections in the past 6 months (aOR: 3.4, 95%CI: 1.9–6.0, *p* < 0.001), ever tested in the hospital (aOR: 5.1, 95%CI: 2.5–10.4, *p* < 0.001), and ever tested in the community (aOR: 1.7, 95%CI: 1.3–2.2, *p* < 0.001; Table 4).

**Table 4:** Factors correlated with self-testing among Chinese female sex workers, 2019.

**Characteristics**		**Syphilis self-tester (*N* = 76)**
	***n*** **(%)**	**cOR (95% CI)**	**aOR (95% CI) ^#^**
**Number of clients served in the past month**			
< =30	53 (69.7)	*ref*	*ref*
31~60	12 (15.8)	0.8 (0.4–1.6)	0.7 (0.3–1.3)
61~90	4 (5.3)	0.6 (0.2–1.7)	0.4 (0.1–1.3)
>90	7 (9.2)	0.6 (0.3–1.4)	0.4 (0.2–1.1)
**Consistently used condom when engaged in commercial vaginal sex in past month**			
Yes	36 (47.4)	1.2 (0.8–2.0)	1.2 (0.8–2.0)
No	40 (52.6)	*ref*	*ref*
**Provided oral sex in the past month**			
Yes	47 (61.8)	1.2 (0.7–1.9)	1.2 (0.7–2.0)
No	29 (38.2)	*ref*	*ref*
**Consistently used condom when engaged in commercial oral sex in past month**			
Yes	7 (14.9)	0.9 (0.4–2.1)	0.6 (0.2–1.5)
No	40 (85.1)	*ref*	*ref*
**Provided anal sex in the past month**			
Yes	25 (32.9)	2.2 (1.4–3.7)^**^	2.6 (1.5–4.3)^***^
No	51 (67.1)	*ref*	*ref*
**Consistently used condom when engaged in commercial anal sex in past month**			
Yes	10 (40.0)	0.9 (0.4–2.2)	0.5 (0.2–1.5)
No	15 (60.0)	*ref*	*ref*
**Used drugs before or during sex**			
Yes	32 (42.1)	2.8 (1.8–4.6)^***^	3.8 (2.3–6.4)^***^
No	44 (57.9)	*ref*	*ref*
**Injected drugs in the past 6 months**			
Yes	3 (3.9)	0.9 (0.3–2.9)	1.1 (0.3–3.8)
No	73 (96.1)	*ref*	*ref*
**Received any kind of HIV/STD-related services in the last year**			
Yes	73 (96.1)	1.1 (0.3–3.6)	1.1 (0.3–3.6)
No	3 (3.9)	*ref*	*ref*
**Bulk purchased condoms**			
Yes	38 (50.0)	0.7 (0.4–1.1)	0.6 (0.4–1.1)
No	38 (50.0)	*ref*	*ref*
**Tested for other STIs in the past 6 months**			
Yes	57 (75.0)	3.2 (1.9–5.4)^***^	3.4 (1.9–6.0)^***^
No	19 (25.0)	*ref*	*ref*
**Diagnosed with other STIs**			
Yes	19 (25.0)	0.7 (0.4–1.3)	0.9 (0.5–1.5)
No	57 (75.0)	*ref*	*ref*
**Ever tested in the hospital**			
Yes	65 (85.5)	3.7 (2.1–6.6)^***^	5.1 (2.5–10.4)^***^
No	11 (14.5)	*ref*	*ref*
**Ever tested in the community**			
Yes	47 (61.8)	1.7 (1.3–2.1)^***^	1.7 (1.3–2.2)^***^
No	29 (38.2)	*ref*	*ref*

** *< 0.01*;

*** *< 0.001*.

# *Multivariate logistic regression adjusted with age, legal marital status, educational attainment, monthly income*.

*cOR, crude odd ratio; aOR, adjusted odd ratio; CI, confidence interval*.

In the subgroup analysis of individuals who had ever used HIV self-testing, three factors were positively correlated with syphilis self-testing: receiving anal sex in the past month (aOR: 1.6, 95%CI: 1.1–2.3, *p* < 0.05), ever tested in the hospital (aOR: 3.3, 95%CI: 1.5–7.5, *p* < 0.01), and ever tested in the community (aOR: 1.3, 95%CI: 1.1–1.7, *p* < 0.001). The following two factors were negatively associated with syphilis self-testing: receiving HIV/STD related services (aOR: 0.4, 95%CI: 0.3–0.6, *p* < 0.001), being diagnosed with other STIs (aOR: 0.6, 95%CI: 0.4–1.0, *p* < 0.05; Supplementary Table S2).

## Discussion

Our study found that syphilis self-testing has the potential of expanding overall syphilis testing uptake, and has limited potential harms among female sex workers. To our knowledge, this study is the first study to examine the syphilis self-testing experience, and associated benefits and harms among female sex workers by collecting data from multiple Chinese provinces. Findings from this study provide insights for the implementation of syphilis prevention programs and research among female sex workers.

Our study showed that quite few Chinese female sex workers used syphilis self-testing. This result is much lower than previously reported among MSM in the Netherlands (13) and China (9). We found that never heard of syphilis self-testing before was the most common reason for not using syphilis self-testing. In the context of wide availability of online syphilis self-testing kits, and the widespread public health and community-based organization programs to promote HIV self-testing (16), more efforts are needed to strengthen the publicity of syphilis self-testing strategy among female sex workers. Integrating syphilis self-testing into existing HIV self-testing services has been shown an acceptable and feasible strategy to promote syphilis self-testing, which could contribute to the dual elimination of HIV and syphilis (17).

Additionally, we found that more than half of syphilis self-testers had never received a syphilis test before, which is consistent with the results among Chinese MSM (9). This indicates that self-testing could increase first-time testing among women that facility-based strategies do not. Our study also observed that those who provided commercial anal sex or used drugs before or during sex are more likely to use syphilis self-testing. This suggests that syphilis self-testing has the potential to reach high-risk female sex workers, which echoes the findings of HIV self-testing studies (18, 19).

Our study found minimal potential harms related to syphilis self-testing among female sex workers. The frequency of pressured testing associated with syphilis self-testing observed in this study is comparable to that previously reported on HIV self-testing among Chinese female sex workers. Although a prior study showed that pressured testing has the benefit of contributing to the subsequent increase of HIV testing frequency among MSM (20), intimate partner violence is still an important concerned factor associated with syphilis self-testing among female sex workers. Our results indicate that syphilis self-testing is not correlated with partner violence. Further research is warranted to evaluate the impact if syphilis self-testing is scaled up.

Our study has several limitations. First, our study used convenience sampling method, which might lead to a selection bias. Although we attempted to recruit a representative sample of female sex workers from different types of venues in various regions of China, generalisability of our results still might be limited. Second, all the data were obtained through self-report, likely resulting in information bias, although we have conducted formative research prior to the study to make the survey more understandable and compatible to the female sex workers. Third, women in this study were recruited from cities which have rich experience of public health outreach programs, which might limited the generalizability of the results to the female sex workers from cities which have fewer syphilis prevention programs.

This study has implications for research and implementation. First, syphilis is a major public health problem, but is often over-looked and under-funded. This study extends the limited evidence that syphilis self-testing could expand syphilis testing among female sex workers, specifically among women who had never tested for syphilis. The ongoing COVID-19 pandemic restrictions on facility-based testing highlight the importance of promoting syphilis self-testing. Second, HIV self-testing has already created extensive infrastructure and widespread public health and community-based organization programs, incorporating syphilis self-testing into the existing HIV self-testing services has the potential to achieve the dual elimination of HIV and syphilis among female sex workers.

## Conclusions

This study demonstrated that syphilis self-testing has the potential to expand syphilis testing coverage and increase testing frequency with limited potential harms among female sex workers. Further evaluation on the intervention effects based on syphilis self-testing among female sex workers are needed.

## Data Availability Statement

The raw data supporting the conclusions of this article will be made available by the authors, without undue reservation.

## Ethics Statement

This study was approved by the Ethics Committee of the Dermatology Hospital of Southern Medical University (2019017). In the formal survey, interested participants who clicked on the survey link were required to sign an informed consent and complete eligibility screening.

## Author Contributions

CW developed the initial concept for the manuscript and drafted an initial manuscript. YW conducted the statistical analysis. XL and BY edited and contributed content to the final draft. All authors have read and approved the final manuscript.

## Funding

This work was supported by Guangdong Medical Research Foundation (A2019402).

## Conflict of Interest

The authors declare that the research was conducted in the absence of any commercial or financial relationships that could be construed as a potential conflict of interest.

## Publisher's Note

All claims expressed in this article are solely those of the authors and do not necessarily represent those of their affiliated organizations, or those of the publisher, the editors and the reviewers. Any product that may be evaluated in this article, or claim that may be made by its manufacturer, is not guaranteed or endorsed by the publisher.

## Abbreviations

FSWs: Female sex workers
MSM: Men who have sex with men
WHO: World Health Organization
HIV: Human immunodeficiency virus
STDs: Sexually transmitted diseases
STI: Sexually transmitted infections
LMIC: Low- and middle- income countries.
