# Supplementary material for: Syphilis Self-Testing Among Female Sex Workers in China: Implications for Expanding Syphilis Screening
Source: Front Public Health. 2022 Apr 13;10:744240. doi: 10.3389/fpubh.2022.744240 (PMC9045586; doi:10.3389/fpubh.2022.744240)
Supplement: Supplementary file 1 [file Data_Sheet_1.docx]

| **Table S1 Difficulties and reasons for performing self-testing among Chinese FSWs** | |
| --- | --- |
| **Items** | **Syphilis self-testing (n, %)** |
| **Difficulties in performing self-testing** | |
| Pricking finger | 28(50.0) |
| Squeezing the finger firmly to extract blood | 16(28.6) |
| Using collection tube to collect blood | 30(53.6) |
| Understanding the instructions for performing the test | 20(35.7) |
| Timing the test | 14(25.0) |
| Removing lancet cap | 9(16.1) |
| Interpreting the results | 13(23.3) |
| **Total** | 56 |
| **Reasons for performing self-testing?** | |
| I wanted to know my infection status | 46(60.5) |
| I want to be the first person to read my test result. | 33(43.4) |
| I had symptoms and I was worried of infection | 24(31.6) |
| I recently had high risk contact | 42(55.3) |
| I was recommended to test by healthcare staff | 12(15.8) |
| My regular partner asked me to test | 12(15.8) |
| A casual partner asked me to test | 4(5.3) |
| **Total** | 76 |
| **Reasons for not using self-testing?** |  |
| Don’t know where to obtain self-test kit | 371(30.6) |
| Haven’t heard of self-testing | 446(36.8) |
| Don’t familiar with instruction guideline | 204(16.9) |
| Have already tested at a clinic | 294(24.3) |
| Worried of giveaway personal information | 223(18.4) |
| Don’t believe the result | 317(26.2) |
| Worried about blood collection | 210(17.3) |
| Worried about not able to interpret the results | 260(21.5) |
| Worried about the cost | 62(5.1) |
| **Total** | 1211 |

**Table S2 Factors correlated with syphilis self-testing among Chinese FSWs who have used HIV self-testing kits, 2019. (N=103)**

| **Characteristics** | **Syphilis self-tester (N=52)** | | |
| --- | --- | --- | --- |
|  | ***n* (%)** | **cOR(95%CI)** | **aOR(95%CI)^#^** |
| **Number of clients served in the past month** |  |  |  |
| <=30 | 34(65.4) | *ref* | *ref* |
| 31~60 | 10(19.2) | 1.3(0.8-2.0) | 1.3(0.8-2.1) |
| 61~90 | 3(5.8) | 1.0(0.4-2.2) | 1.0(0.3-2.7) |
| >90 | 5(9.6) | 0.6(0.3-1.2) | 0.4(0.2-0.9) |
| **Consistently used condom when engaged in commercial vaginal sex in past month** | | | |
| Yes | 23(44.2) | 1.3(0.9-1.9) | 1.1(0.7-1.6) |
| No | 29(55.8) | *ref* | *ref* |
| **Provided oral sex in the past month** |  |  |  |
| Yes | 36(69.2) | 1.2(0.8-1.8) | 1.2(0.7-1.9) |
| No | 16(30.8) | *ref* | *ref* |
| **Consistently used condom when engaged in commercial oral sex in past month** | | | |
| Yes | 5(13.9) | 0.8(0.4-1.5) | 0.7(0.3-1.3) |
| No | 31(86.1) | *ref* | *ref* |
| **Provided anal sex in the past month** |  |  |  |
| Yes | 21(40.4) | **1.5(1.0-2.2)*** | **1.6(1.1-2.3)*** |
| No | 31(59.6) | *ref* | *ref* |
| **Consistently used condom when engaged in commercial anal sex in past month** | | | |
| Yes | 10(47.6) | 1.3(0.8-2.2) | 1.1(0.7-1.7) |
| No | 11(52.4) | *ref* | *ref* |
| **Used drugs before or during sex** |  |  |  |
| Yes | 21(40.4) | 1.2(0.8-1.8) | 1.2(0.8-1.7) |
| No | 31(59.6) | *ref* | *ref* |
| **Injected drugs in the past 6 months** |  |  |  |
| Yes | 3(5.8) | 0.7(0.3-1.8) | 0.6(0.3-1.5) |
| No | 49(94.2) | *ref* | *ref* |
| **Received any kind of HIV/STD-related services in the last year** | | | |
| Yes | 50(96.2) | **0.5(0.4-0.6)***** | **0.4(0.3-0.6)***** |
| No | 2(3.8) | *ref* | *ref* |
| **Bulk purchased condoms** |  |  |  |
| Yes | 29(55.8) | 0.8(0.5-1.1) | 0.8(0.5-1.2) |
| No | 23(44.2) | *ref* | *ref* |
| **Tested for other STIs in the past 6 months** |  |  |  |
| Yes | 42(80.8) | 1.4(0.8-2.4) | 1.4(0.8-2.5) |
| No | 10(19.2) | *ref* | *ref* |
| **Diagnosed with other STIs** |  |  |  |
| Yes | 13(25.0) | **0.6(0.4-0.9)*** | **0.6(0.4-1.0)*** |
| No | 39(75.0) | *ref* | *ref* |
| **Ever tested in the hospital** |  |  |  |
| Yes | 48(92.3) | **3.2(1.5-6.9)**** | **3.3(1.5-7.5)**** |
| No | 4(7.7) | *ref* | *ref* |
| **Ever tested in the community** |  |  |  |
| Yes | 35(67.3) | **1.5(1.2-1.8)***** | **1.3(1.1-1.7)***** |
| No | 17(32.7) | *ref* | *ref* |

*<0.05; **<0.01; ***<0.001.

#Multivariate logistic regression adjusted with age, legal marital status, educational attainment, monthly income

cOR: crude odd ratio; aOR: adjusted odd ratio; CI: confidence interval.
